# BeEAM High-Dose Chemotherapy with Polatuzumab (Pola-BeEAM) before ASCT in Patients with DLBCL—A Pilot Study

**DOI:** 10.3390/jcm11133748

**Published:** 2022-06-28

**Authors:** Tanja Stoffel, Ulrike Bacher, Yara Banz, Michael Daskalakis, Urban Novak, Thomas Pabst

**Affiliations:** 1 Department of Medical Oncology, Bern University Hospital, University of Bern, 3010 Bern, Switzerland; tanja.stoffel@students.unibe.ch (T.S.); urban.novak@insel.ch (U.N.); 2 Department of Hematology, Central Hematology Laboratory, Inselspital, Bern University Hospital, University of Bern, 3010 Bern, Switzerland; veraulrike.bacher@insel.ch (U.B.); michael.daskalakis@insel.ch (M.D.); 3 Institute of Pathology, University of Bern, 3010 Bern, Switzerland; yara.banz@pathology.unibe.ch

**Keywords:** Pola-BeEAM, polatuzumab vedotin, bendamustine, autologous stem cell transplantation, high-dose chemotherapy, conditioning regimen, DLBCL

## Abstract

(1) Introduction: BEAM is a high-dose chemotherapy (HDCT) frequently administered before autologous stem cell transplantation (ASCT) in diffuse large B-cell lymphoma (DLBCL). Bendamustine replacing BCNU (BeEAM) is similarly effective at lower toxicities. However, relapse remains the major cause of death in DLBCL. (2) Methods: This is a 12-patient pilot study of the BeEAM preparative regimen with additional polatuzumab vedotin (PV, targeting CD79b) aiming to establish feasibility and to reduce toxicity without increasing the early progression rate. PV was given once at the standard dose of 1.8 mg/kg at day −6 together with BeEAM-HDCT (days −7 to −1) before ASCT. (3) Results: 8/12 patients (67%) received PV with BeEAM as a consolidation of first-line treatment, and 4/12 patients (33%) received PV with BeEAM after relapse treatment. All patients experienced complete engraftment (neutrophils: median 11 days; platelets: 13 days). Gastrointestinal toxicities occurred in 7/12 patients (58%, grade 3). All patients developed neutropenic infections with at least one identified pathogen (bacterial: 10/12 patients; viral: 2/12; and fungal: 1/12). The complete remission rate by PET-CT 100 days post-ASCT was 92%, with one mortality due to early progression. Eleven out of twelve patients (92%) were alive without progression after a median follow-up of 15 months. (4) Conclusions: Our study with 12 patients suggests that combining PV with BeEAM HDCT is feasible and safe, but the limited cohort prevents definite conclusions regarding efficacy. Larger cohorts must be evaluated.

## 1. Introduction

Despite significant improvement in recent decades, the treatment of patients with refractory or relapsing diffuse large B-cell lymphoma (DLBCL) remains challenging [1,2,3,4,5]. Numerous studies have reported that high-dose chemotherapy (HDCT) followed by autologous stem cell transplantation (ASCT) increases event-free (EFS), progression-free (PFS) and overall survival (OS) compared to conventional chemotherapy in relapsed DLBCL patients, thereby representing for decades the preferred consolidation approach for patients with chemosensitive DLBCL after first relapse [6,7,8,9].

The most commonly applied conditioning regimen before ASCT is the BEAM regimen, comprising BCNU, etoposide, cytarabine and melphalan [10,11]. However, with regard to issues such as idiopathic pneumonia syndrome (IPS), delivery shortages and the rising costs of BCNU, several studies suggested replacing BCNU with bendamustine [11,12,13]. Bendamustine combined with BEAM (BeEAM) was shown to have an acceptable safety profile and a comparable PFS and OS to BEAM [11,12]. Nevertheless, progression of the underlying disease remains the major cause of death in relapsing DLBCL patients, and improved efficacy of HDCT is an unmet need. Here, we performed a pilot study combining PV with BeEAM in 12 patients with DLBCL undergoing ASCT, with eight cases being a consolidation of the first response.

Currently, chimeric antigen t-cell therapy (CAR-T) as second-line treatment shows promising results for DLBCL patients, but this option awaits validation; therefore, HDCT with ASCT remains the standard treatment in chemosensitive DLBCL patients for achieving a second remission [14,15,16,17,18]. The combination of immunotherapy together with BeEAM HDCT is an obvious approach given the usually good tolerance of antibody compounds, without adding significant additional toxicity. However, data are missing so far regarding whether the combination of immunotherapy together with HDCT improves outcomes. Polatuzumab vedotin (PV) is a promising candidate to be combined with the BeEAM conditioning regimen (Pola-BeEAM) to further improve the efficacy of HDCT in patients with chemosensitive DLBCL. PV is an antibody-drug conjugate (ADC) consisting of the monoclonal anti-CD79b antibody conjugated by a linker sequence with the cytotoxic monomethyl auristatin E (MMAE) compound. Once PV binds to the CD79b receptor, it is internalized toward the lysosome. Through cleavage of the linker via lysosomal proteases, MMAE is activated, ultimately leading to apoptosis of the target B-cell [19].

PV combined with bendamustine and rituximab (PV-BR) is approved for the treatment of patients with transplant-ineligible relapsed or refractory B-cell lymphomas [20]. This pivotal phase 2 study and subsequent reports have demonstrated better complete remission (CR), PFS and OS compared to bendamustine and rituximab (BR) alone as well as an overall good safety profile [20,21]. Based on these results, we tested PV in combination with HDCT in 12 DLBCL patients prior to ASCT. Specifically, we investigated for the first time the addition of a single standard dose of PV together with full dose BeEAM conditioning in 12 DLBCL patients. The aim was to evaluate the feasibility and safety of the Pola-BeEAM regimen prior to ASCT. This is the first study to use PV as part of a HDCT regimen in a cohort of 12 patients with DLBCL.

## 2. Methods

### 2.1. Patients

In this single-center study, 12 patients consecutively diagnosed with DLBCL were analyzed for the feasibility and safety of HDCT using the Pola-BeEAM regimen before ASCT. All patients were treated at the Department of Medical Oncology, University Hospital Berne, Switzerland, between August 2020 and March 2021. The patient characteristics are summarized in Table 1. The study was approved by a decision of the local ethics committee in Bern, Switzerland (decision number #2021-00442).

### 2.2. Assessments and Definitions

DLBCL was diagnosed based on molecular pathological investigations and classified according to the Hans algorithm. Staging of lymphoma was according to the Ann Arbor classification and the International Prognostic Index (IPI) risk score [22]. CT, PET-CT and/or MRI were applied for staging and response assessment. Bone marrow aspiration and cerebrospinal fluid investigation were used for the diagnosis of infiltration of the bone marrow or central nervous system (CNS), respectively. Lymphoma manifestation exceeding a diameter of 7 cm was classified as bulky. The Eastern Cooperative Oncology Group (ECOG) performance status [23] as well as the Karnofsky score [24] were used for the functional impairment assessment of patients. Toxicities were graded according to the Common Terminology Criteria for Adverse Events v.5.0 (CTCAE) [25].

### 2.3. Treatment

Mobilization was performed using granulocyte-colony stimulating factor (G-CSF) with combined chemotherapy in all patients. After mobilization, peripheral stem cells were collected; no bone marrow collection was performed. On days −7 and −6 before ASCT, patients received 200 mg/m^2^ bendamustine diluted in 500 mL 0.9% NaCl for 2 h. A single dose of 140 mg PV diluted in 100 mL of 5% glucose was given on day −6 over 90 min. On days −5 to −2, 150 mg/m^2^ etoposide and 200 mg/m^2^ cytarabine, both diluted in 500 mL 0.9% NaCl, were consecutively administered over 30 min, in a 12 h interval twice daily. On day −1 before ASCT, 140 mg/m^2^ melphalan diluted in 500 mL 0.9% NaCl was infused in 60 min. To prevent renal impairment, patients were hydrated prior to bendamustine administration with 1000 mL 0.9% NaCl for two hours, followed by 20 mg furosemide iv and 1000 mL 0.9% NaCl for one hour after bendamustine. In addition, 1000 mL 0.9% NaCl was given for the next three hours, and additionally, 1000 mL 0.9% NaCl was given for the next 12 h. Hydration before etoposide and before melphalan was according to standard institutional procedures.

To avoid PV-related transfusion reactions, 200 mg of the histamine H2 inhibitor cimetidine and 2 mg of clemastine were given 30 min prior to PV infusion, together with oral 1000 mg paracetamol. Supportive treatment was given according to internal guidelines for HDCT/ASCT. Patients received filgrastim Teva^®^ starting from day +6 and maintained up to day +12. In addition, patients received 5 mg of folic acid daily. During treatment, patients were hospitalized in one- or two-bed rooms without isolation. Patients routinely received antiviral (oral acyclovir 500 mg twice daily) and antifungal prophylaxis (oral fluconazole 400 mg once weekly), and oral sulfamethoxazole/trimethoprim 800/160 mg three times per week. Sulfamethoxazole/trimethoprim administration started on day +1. Corticosteroids were administered as engraftment-syndrome prophylaxis from day +8 to day +12 and low vitamin D levels were supplemented [26,27]. Patients received platelet and red blood cell transfusions when platelets fell below 10 G/L (or if clinically indicated in case of bleeding or for interventions) and hemoglobin was below 80 g/L, respectively. In case of red blood cell transfusions, a single package (275 mL) was administered. Febrile episodes were defined as recurrence of fever verified after the identification of a novel infectious pathogen. Patients were hospitalized for the entire procedure, starting with the first day of HDCT, and they were dismissed after hematologic recovery and adequate physical reconditioning.

## 3. Results

### 3.1. Patients

Details of patient characteristics at diagnosis and at ASCT are listed in Table 1 and in Supplementary Table S1. The cohort comprised 12 patients with a gender ratio of 3:1 (m/f) and a median age of 61 years. All patients were diagnosed with DLBCL. Bone marrow and CNS infiltration were identified in one patient each. The median time from first diagnosis of lymphoma until HDCT was 6 months. An overview of the treatment lines given before Pola-BeEAM HDCT is summarized in Table 1. In Table 2, details on each patient’s treatment prior to ASCT are presented.

After first line therapy, eight patients were in CR (of which two relapsed), two were in partial remission (PR), and two patients were primary refractory. One of the two patients in PR received ibrutinib during first-line therapy as part of the SAKK35/14 study. Two patients relapsed after they achieved CR after first-line treatment. Six of the twelve patients received ASCT as part of the first-line consolidation given their high-risk presentation at first diagnosis. At the time of ASCT, ten patients were in CR and two patients were in PR.

### 3.2. Pola-BeEAM High-Dose Treatment

The source of autologous stem cells was peripheral blood in all patients. No CD34+ selection was performed. We aimed for at least 2.0 × 10^6^ CD34+ cells/kg b.w. during apheresis. After the administration of Pola-BeEAM, a median of 4.1 × 10^6^ CD34+ cells/kg b.w. were transplanted. Details on the HDCT, engraftment, infections and toxicities are summarized in Table 3. No PV-related transfusion reactions were observed, and prophylactic medication was given in all patients, as planned. HDCT was given in all patients at the planned dose and without delay due to eventual side effects. The median duration of total hospitalization for the Pola-BeEAM HDCT treatment was 23 days (range 20–34 days).

### 3.3. Hematologic Recovery

A median of three erythrocyte concentrates and a median of six platelet concentrates were administered. All patients had successful hematologic engraftment (Table 3). The median time to recovery of platelets above 20 G/L was 13 days (range 10–25) after ASCT. An absolute neutrophil count (ANC) above 0.5 G/L was achieved after a median of 11 days (range 10–13) after ASCT. Lymphocytes recovered above 1.0 G/L after a median of 25 days (range 16–51).

### 3.4. Infections during Hospitalization

The antibiotic treatment triggered by a febrile episode and consecutive search for a causative pathogen was documented in all patients. Pathogens responsible for the febrile episode were identified in eleven patients (92%). Gram-positive bacterial infections were the most common (75%), followed by gram-negative infections (42%), and viral (17%) and fungal infections (8%). The details are listed in Table 3.

### 3.5. Non-Hematologic Toxicities

Toxicities were classified according to the CTCAE v.5.0 manual. Overall, ten patients (83%) experienced toxicities grade 3 or higher. Toxicities, as expected from the BeEAM HDCT treatment, predominantly affected the oral and gastrointestinal mucosa, with the most common toxicities being mucositis (100%), diarrhea (92%), dysphagia (75%) and neutropenic colitis (58%). Acute kidney injury occurred in two patients. No peripheral sensory neuropathy was observed. In general, toxicities were manageable. One patient needed to be admitted to the intensive care unit (ICU) due to septic shock.

### 3.6. Outcome

For the evaluation of safety and tolerance of the Pola-BeEAM conditioning regimen, data on late toxicities and the response 100 days after administration of the HDCT and at last follow-up were collected and are summarized in Table 4. Following discharge after HDCT hospitalization, no re-hospitalization occurred due to infectious complications or late toxicities. One patient died before the assessment at 100 days due to early progression of the lymphoma. The remaining eleven patients achieved or maintained a CR 100 days after Pola-BeEAM treatment. After a median follow-up of 15 months after Pola-BeEAM HDCT, none of the remaining eleven patients had relapsed or died, accounting for a PFS and OS of 92%.

## 4. Discussion

In this pilot study, we evaluated the feasibility, safety and tolerance of adding a single dose of the monoclonal CD79b antibody PV given together with BeEAM HDCT in 12 patients with DLBCL undergoing ASCT. A significant proportion of these patients were receiving HDCT as a consolidation of the first-line treatment due to high-risk presentation at initial diagnosis. Patients were considered eligible for ASCT in this study based on standard criteria such as stage IV disease (n = 6), refractory disease (n = 2), partial response (n = 2) or relapse after first-line treatment (n = 2). Out of the twelve patients, two were in PR and ten were in CR when they received ASCT. First, we found that the combination of PV with BeEAM was both feasible and safe. All patients had successful hematologic engraftment with a median interval of 11 days for neutrophils >0.5 G/L and a median of 13 days for thrombocytes >20 G/L. Additionally, no treatment-related deaths were observed in our study.

Our results were comparable with those from our previous study [12] in which we evaluated the BeEAM regimen (without any B-cell antibody) prior to ASCT in 39 DLBCL patients with a similar median age at initial diagnosis (60 years versus 61 years) [12]. In particular, we reported similar results in our previous work [12] with regard to hematologic recovery as compared to this study. When compared to other studies such as Hahn et al. [11], Chantepie et al. [13] and Visani et al. [28], where BeEAM was applied predominantly in relapsed or refractory lymphoma patients, the hematological recovery was similar, ranging from a median of 11–13 days for thrombocytes >20 G/L to 10–11.7 days for neutrophils >0.5 G/L. Of particular interest is the analysis of the types and severity of infections following Pola-BeEAM HDCT, given the potential immunosuppressive effect of PV. In this cohort of 12 patients, we observed an expected range of bacterial, viral and fungal infections with an incidence and severity commonly observed in HDCT patients. The infection rates in this study were higher compared to our previous results (92% after Pola-BeEAM and 79% after BeEAM) [12] and those of other studies reporting on the BeEAM regimen without PV, i.e., Hahn et al. (70.7%) [11], Chantepie et al. (78.2%) [13] or Visani et al. [28], where in only 7% of patients an infection was documented [28]. However, a comparison of the different studies must be performed with caution.

Fever in aplasia occurred in all patients (100%), similarly to our previous study (100%) or to Hahn et al. (100%) [11,12]. Visani et al. reported lower rates of fever in aplasia, with 51% of patients [28]. Fever is a frequent side effect of BeEAM treatment. Saleh et al. applied BeEAM on relapsed/refractory non-Hodgkin lymphoma (NHL) patients with a control BEAM counterpart paired cohort and found a higher number of days with fever compared to BEAM [29]. The results from our study and those from the aforementioned studies are difficult to compare, especially due to the different patient characteristics. BeEAM without PV was mostly tested in relapsed or refractory lymphoma patients, whereas in this study, Pola-BeEAM was applied mostly to DLBCL patients who received ASCT as a first-line consolidation. Due to the small size of our cohort, the results should be interpreted cautiously. Larger cohorts will be needed to identify possible preferential infectious complications related to the addition of PV to the BeEAM regimen.

We observed a high rate of grade III gastrointestinal toxicities of 58% (7/12 patients), in our PV-BeEAM cohort, which is a characteristic of BeEAM alone. Saleh et al. presented higher rates of high-grade diarrhea in BeEAM patients compared to the BEAM patients [29]. Looking at the incidence of gastrointestinal toxicities in other studies such as Chantepie et al. and Visani et al., where BeEAM was used in relapsed and refractory lymphoma patients, the incidence of high-grade toxicities was a little lower than in our cohort [13,28]. For instance, Hahn et al. [11] reported a mucositis rate of 88% as compared to 100% in our present cohort and neutropenic colitis in 27% as compared to 58% in our study. In addition, the BeEAM regimen is characterized by an increased incidence of acute renal toxicities (acute kidney injuries, AKI) [30]. In our cohort, again, we observed two (17%) fully reversible AKI grade 3, which is similar to the data previously reported [11,12,13,30]. Once again, it is important to mention that this study is unable to directly compare the toxicological profiles of BeEAM and Pola-BeEAM. Interestingly, we did not observe the occurrence of peripheral sensory neuropathy in our cohort. However, no patient had preexisting peripheral sensory neuropathy. The first occurrence of peripheral sensory neuropathy was reported in a study by Sehn et al. in 43.6% of the patients at grade 1 or 2 after PV in combination with bendamustine and rituximab [20].

After a median follow-up of 15 months, one patient (8%) relapsed and died due to progression so far, leading to a PFS and an OS of 92%. Compared to our previous studies on BeEAM alone with a PFS of 69% and an OS of 72% in the 2-year follow-up [12], these results seem promising. Additionally, compared to other studies such as Hahn et al. (3-year OS 71%, 3-year PFS 74.1%) or Visani et al. (81% in CR after 18 months of follow-up), where BeEAM was applied on relapsed and refractory lymphoma patients, the PFS and OS of this study are encouraging [11,28]. However, the limited number of patients and the limited follow-up in our study do not allow us to draw definite conclusions on the efficacy of the Pola-BeEAM regimen, and the inclusion of a high proportion of patients transplanted with lymphoma in remission after first-line therapy may also account for the good results. Thus, the heterogenous composition of the study cohort (including patients with high-risk stage IV disease transplanted in first CR) renders definite conclusions of the efficacy of the novel conditioning regimen impossible. Larger clinical trials with a longer follow-up are needed.

In conclusion, our data from this pilot study with twelve patients suggest that Pola-BeEAM is a feasible and safe conditioning regimen for patients with DLBCL, and a longer follow-up appears warranted. Larger randomized clinical trials including patients with refractory/relapsed DLBCL are needed to fully elucidate the outcomes and toxicities after Pola-BeEAM treatment, particularly when used in patients with relapsed or primary refractory DLBCL.

## Figures and Tables

**Table 1:** Patient characteristics and previous treatments.

Parameter	Results
Age at first diagnosis, median, year (range)	61 (37–73)
Gender, female/male, *n* (%)	3/9 (25/75%)
**Histology, *n* (%)**	
De novo DLBCL	7/12 (58%)
Transformed DLBCL	5/12 (42%)
from follicular lymphoma	4/12 (34%)
from marginal zone lymphoma	1/12 (8%)
GCB type	4/12 (33%)
ABC type	8/12 (67%)
**IPI, *n* (%)**	
2	1/12 (8%)
3	4/12 (34%)
4–5	7/12 (58%)
Bone marrow infiltration, *n* (%)	1/12 (8%)
CNS infiltration, *n* (%)	1/12 (8%)
Bulky disease, *n* (%)	4/12 (34%)
B-symptoms, *n* (%)	2/12 (17%)
Time from first diagnosis to HDCT, median, months (range)	6 (4–63)
Previous lines of therapy before HDCT, median, *n* (range)	2 (1–5)
Primary refractory DLBCL	2/12 (17%)
PR after first line of therapy	2/12 (17%)
CR after first line of therapy	8/12 (67%)
Relapsed DLBCL	2/12 (17%)
CR duration shorter than one year	2/12 (17%)
**Previous therapies (*n*)**	
***First line therapy***	
R-CHOP	9
R-DA-EPOCH	1
R-DHAO	1
Ibrutinib	1
Additional radiotherapy, *n* (%)	2/12 (17%)
***Second line therapy***	
R-DHAP	3
R-DHAO	2
R-CHOP	1
R-CODOX-M/R-IVAC	1
Additional radiotherapy, *n* (%)	1/12 (8%)
***Third line therapy***	
R-GDP	1
Additional radiotherapy, *n* (%)	0
**Remission status prior to HDCT, *n* (%)**	
CR	10/12 (83%)
PR	2/12 (17%)

***DLBCL*** *: diffuse large B-cell lymphoma, **GCB:** germinal center B-cell, **ABC:** activated B cell type, **IPI**: international prognostic index, **CNS**: central nervous system; **PR**: partial remission; **CR**: complete remission; **R**: rituximab, **CHOP**: cyclophosphamide, doxorubicin, vincristine, prednisone, **DHAP**: dexamethasone, high-dose cytarabine, cisplatin, **DHAO**: dexamethasone, high-dose cytarabine, oxaliplatin, **DA-EPOCH**: dose-adjusted etoposide, prednisone, vincristine, cyclophosphamide, doxorubicin, **CODOX**: cyclophosphamide, cytarabine, vincristine, doxorubicin, **IVAC**: ifosfamide, etoposide, cytarabine, **GDP**: gemcitabine, dexamethasone, cisplatin.*

**Table 2:** Details on patients’ previous treatments.

Patient Number	Sex	Age at First Diagnosis	1st Line of Therapy	RT	Response	2nd Line of Therapy	RT	Response	3rd Line of Therapy	Response	Reason for 1st-Line ASCT	Relapse after ASCT	Death after ASCT
4296680	m	37	6 cycles of R-DA-EPOCH	1	PR	3 × R-DHAP, 3 × MTX	1	CR			n.a.	No	No
16007387	m	67	6 × R-CHOP		CR						Stage IV disease at diagnosis	No	No
12784362	m	47	6 × R-CHOP + 3 × MTX		PR	2 × R-DHAP, 1 cycle of R-DHAO		PR			n.a.	No	No
3032167	m	73	6 × R-CHOP		CR						Stage IV disease at diagnosis	No	No
9981667	m	63	3 × R-CHOP	1	CR, followed by relapse	3 × R-DHAO		CR			n.a.	No	No
16050304	f	63	6 × R-CHOP		CR						Stage IV disease at diagnosis	No	No
3333019	f	59	6 × R-CHOP		CR, followed by relapse	3 × R-DHAP		CR			n.a.	No	No
15919544	m	59	ibrutinib + rituximab		Refractory	6 × R-CHOP		CR			n.a.	No	No
9997814	f	67	6 × R-CHOP		CR						Stage IV disease at diagnosis	Yes	Yes (at day +26)
16261933	m	62	6 × R-CHOP		CR						Stage IV disease at diagnosis	No	No
16415060	m	60	3 × R-DHAO		CR						Stage IV disease at diagnosis	No	No
16278372	m	59	1 × R-CHOP		Refractory	2 × R-CODOX-M and R-IVAC each		PR	3 × R-GDP	PR	n.a.	No	No

***RT:*** *radiotherapy; **ASCT**: autologous stem cell transplantation; **PR**: partial remission; **n.a.**: not available; **CR:** complete remission; **R**: rituximab; **DA-EPOCH**: dose-adjusted etoposide, prednisone, vincristine, cyclophosphamide, doxorubicin; **DHAP**: dexamethasone, high-dose cytarabine, cisplatin; **CHOP**: cyclophosphamide, doxorubicin, vincristine, prednisone; **DHAO**: dexamethasone, high-dose cytarabine, oxaliplatin; **CODOX**: cyclophosphamide, cytarabine, vincristine, doxorubicin; **IVAC**: ifosfamide, etoposide, cytarabine; **GDP**: gemcitabine, dexamethasone, cisplatin; **MTX**: methotrexate.*

**Table 3:** Details of high-dose chemotherapy, engraftment, infections, and hematological- and non-hematological toxicities.

Parameter	Results	
Apheresis from peripheral blood, *n* (%)	12/12 (100%)	
Pola-BeEAM administered at full dose, *n* (%)	12/12 (100%)	
PV-associated transfusion reactions, *n* (%)	0/12 (100%)	
Transplanted CD34+ cells, median, ×10^6^/kg b.w. (range)	4.1 (2.6–7.5)	
**Median time to engraftment, days (range)**		
Tc > 20 G/L	13 (10–25)	
Tc > 50 G/L	19 (13–51)	
Tc > 100 G/L	31 (14–51)	
Neutrophils > 0.5 G/L	11 (10–13)	
Neutrophils > 1.0 G/L	11 (9–12)	
Lymphocytes > 1.0 G/L	25 (16–51)	
Hospitalization, median, days (range)	23 (20–34)	
TPN given, *n* (%)	11 (92%)	
Units of erythrocyte transfusions, median, *n* (range)	3 (0–10)	
Units of platelet transfusions, median, *n* (range)	6 (2–15)	
Weight changes, median, kg (range)	−2 (−8; +3)	
**Infections**		
At least one febrile episode, *n* (%)	12/12 (100%)	
Median number of febrile episodes, *n* (range)	2 (1–3)	
Median days with fever, *n* (range)	5 (2–24)	
Patients with at least one causative identified pathogen, *n* (%)	11/12 (92%)	
**Bacterial, *n* (%)**	10/12 (83%)	
Patients with gram+ bacteria identified	9/12 (75%)	
Patients with gram- bacteria identified	5/12 (42%)	
**Viral, *n* (%)**	2/12 (17%)	
**Fungal, *n* (%)**	1/12 (8%)	
Antibiotics given, *n* (%)	12/12 (100%)	
**Non-hematological toxicities**		
Patients with toxicities, all grades, *n* (%)	12/12 (100%)	
Patients with >1 toxicity, all grades, *n* (%)	12/12 (100%)	
Patients with grade 3–4 toxicities, *n* (%)	10/12 (83%)	
**Grades of toxicities, *n***	**Grade 1–2**	**Grade 3–4**
Mucositis	8	4
Diarrhea	7	4
Dysphagia	8	1
Neutropenic colitis	7	0
Acute kidney injury	0	2
Gastrointestinal bleeding	0	1
Thromboembolic events	0	1
Atrial fibrillation	1	0
**ICU admission, *n***	1	0
Due to septic shock	1	0

***Pola-BeEAM:*** *polatuzumab vedotin, bendamustine, etoposide, cytarabine, melphalan, **PV:** polatuzumab vedotin, **Tc**: thrombocytes, **TPN**: total parenteral nutrition, **ICU**: intensive care unit.*

**Table 4:** Outcome.

Parameter, *n* (%)	Results
CR 100 days after ASCT	11/12 (92%)
Progression before 100 days after ASCT	1/12 (8%)
Relapse during follow-up	1/12 (8%)
Death during follow-up *	1/12 (8%)
Secondary malignancies after ASCT	0/12 (0%)

CR: complete remission; ASCT: autologous stem cell transplantation. * at day +26 following ASCT.

## Data Availability

Data are available from the corresponding author by email request.

## Supplementary Materials

The following supporting information can be downloaded at: https://www.mdpi.com/article/10.3390/jcm11133748/s1, Table S1: Details on patients’ previous treatments.
