# Supplementary material for: BeEAM High-Dose Chemotherapy with Polatuzumab (Pola-BeEAM) before ASCT in Patients with DLBCL—A Pilot Study
Source: J Clin Med. 2022 Jun 28;11(13):3748. doi: 10.3390/jcm11133748 (PMC9267272; doi:10.3390/jcm11133748)
Supplement: Supplementary file 1 [file jcm-1713485-supplementary.pdf › jcm-1713485-supplementary.pdf]

Supplementary Table S1: Details on patients' previous treatments.

Patient number	sex	age at first diagnosis	First line of therapy	RT	Response	Second line of Therapy	RT	Response	Third line of therapy	RT	Response
4296680	m	37	6 cycles of R-DA-EPOCH	1	PR	3 cycles of R-DHAP, 3 cycles of Methotrexate	1	CR			
16007387	m	67	6 cycles of R-CHOP		CR						
12784362	m	47	6 cycles of R-CHOP + 3 cycles of methotrexate		PR	2 cycles of R-DHAP, 1 cycle of R-DHAO		PR			
3032167	m	73	6 cycles of R-CHOP		CR						
9981667	m	63	3 cycles of R-CHOP	1	CR	3 cycles of R-DHAO		CR			
16050304	f	63	6 cycles of R-CHOP		CR						
3333019	f	59	6 cycles of R-CHOP		CR	3 cycles of R-DHAP		CR			
15919544	m	59	Ibrutinib		Refractory	6 cycles of R-CHOP		CR			
9997814	f	67	6 cycles of R-CHOP		CR						
16261933	m	62	6 cycles of R-CHOP		CR						
16415060	m	60	3 cycles of R-DHAO		CR						
16278372	m	59	1 cycle of R-CHOP		Refractory	2 cycles of R-CODOX-M and R-IVAC each		PR	3 cycles of R-GDP		PR

RT: *radiotherapy, PR: partial remission* ***CR:*** *complete remission,* ***R****: rituximab,* ***DA-EPOCH****: dose adjusted etoposide, prednisone, vincristine, cyclophosphamide, doxorubicin,* ***DHAP****: dexamethasone, high-dose cytarabine, cisplatin,* ***CHOP****: cyclophosphamide, doxorubicin, vincristine, prednisone,* ***DHAO****: dexamethasone, high-dose cytarabine, oxaliplatin,* ***CODOX****: cyclophosphamide, cytarabine, vincristine, doxorubicin,* ***IVAC****: ifosfamide, etoposide, cytarabine,* ***GDP****: gemcitabine, dexamethasone, cisplatin.*
